# Supplementary material for: Trends in cost and consumption of essential medicines for non-communicable diseases in Azerbaijan, Georgia, and Uzbekistan, from 2019 to 2021
Source: PLoS One. 2023 Dec 7;18(12):e0294680. doi: 10.1371/journal.pone.0294680 (PMC10703197; doi:10.1371/journal.pone.0294680)
Supplement: S1 File — (DOCX) [file pone.0294680.s001.docx]

## Appendix 1. Key parameters of health systems and pharmaceutical pricing systems in Azerbaijan, Georgia, and Uzbekistan.

*Key parameters of health systems and pharmaceutical pricing systems in Azerbaijan, Georgia, and Uzbekistan*

|  | **Azerbaijan** | **Georgia** | **Uzbekistan** |
| --- | --- | --- | --- |
| Population(1) | 2019 - 10,024,283  2020 - 10,093,121  2021 - 10,145,212 | 2019 - 3,720,161  2020 - 3,722,716  2021 - 3,708,610 | 2019 - 33,580,350  2020 - 34,232,050  2021 - 34,915,100 |
| Average monthly salary (2021) | $416.10 | $356.00 | $295.84 |
| Health system | National Health Service | National Health Service | National Health Service |
| The current health expenditure (CHE) per capita, US$ (2019)(2) | $193.1 | $291.1 | $98.6 |
| Out-of-pocket expenditure (OOP) as percent of CHE (2019)(2) | 67.96% | 46.77% | 57.68% |
| Spending for pharmaceuticals in retail network, per capita (calculated as part of this analysis) | 2019 - $39.33  2020 -$43.20  2021 - $45.98 | 2019 - $105.97  2020 - $69.78  2021 - $90.68 | 2019 -$31.43  2020 - $37.86 |
| Spending for pharmaceuticals (prescribed and over-the-counter) as percent of private health expenditure (2019) | 30% | 62% | 55% |
| Price regulation(3,4) | External price referencing and internal price referencing used for fixed registered prices and regulated marginal pharmacy and wholesaler mark-ups for medicines in outpatient settings | No price regulation system | Transition from fixed prices to regulated mark-ups to ERP |
| Use of National Essential Medicines List | NEML containing 305 INNs | No NEML | NEML containing 430 INNs |
| Reimbursement list for medicines in outpatient settings(3) | List of “vital medicines” for defined diseases | >100 medicines reimbursed, depending on disease, and the social status and age of the patient | List for medicines for 13 defined “socially significant” diseases |
| Peak incidence of COVID-19(5) | Q4 2020 Q2 2021, Q3 2021, Q4 2021 | Q4 2020 Q3 2021, Q4 2021 | Q3 2020, Q2 2021 |

**Figure 1. Disability-adjusted life years lost per 100,000 people.**

Data from IHME(6).

**Figure 2. Prevalence per 100,000 people.**

Data from IHME(6).

**Figure 3. Age-standardized death rate per 100,000 people.**

Data from IHME(6).

**References**

1. World Bank. Population, total - Azerbaijan, Georgia, Uzbekistan [Internet]. Available from: https://data.worldbank.org/indicator/SP.POP.TOTL?locations=AZ-GE-UZ&view=chart

2. World Health Organization. The Global Health Expenditure database [Internet]. Available from: https://apps.who.int/nha/database/ViewData/Indicators/en

3. WHO Regional Office for Europe. Pharmaceutical pricing and reimbursement systems in Eastern Europe and Central Asia. Pharmaceutical pricing and reimbursement information report. [Internet]. 2020. Available from: https://www.euro.who.int/__data/assets/pdf_file/0007/455938/Pharmaceutical-pricing-eng.pdf

4. World Health Organization. Health systems in action: Uzbekistan [Internet]. 2021. Available from: https://apps.who.int/iris/handle/10665/349236

5. World Health Organization. WHO Coronavirus (COVID-19) Dashboard [Internet]. Available from: https://covid19.who.int/

6. Vos T, Lim SS, Abbafati C, Abbas KM, Abbasi M, Abbasifard M, et al. Global burden of 369 diseases and injuries in 204 countries and territories, 1990–2019: a systematic analysis for the Global Burden of Disease Study 2019. The Lancet. 2020 Oct;396(10258):1204–22.
