# Supplementary material for: Trends in cost and consumption of essential medicines for non-communicable diseases in Azerbaijan, Georgia, and Uzbekistan, from 2019 to 2021
Source: PLoS One. 2023 Dec 7;18(12):e0294680. doi: 10.1371/journal.pone.0294680 (PMC10703197; doi:10.1371/journal.pone.0294680)
Supplement: S2 File — (DOCX) [file pone.0294680.s002.docx]

## Appendix 2. Accessibility of medicines used in NCDs in Azerbaijan, Georgia, and Uzbekistan, 2019-2021.

### Appendix 2. Section 1. Medicines for CVD treatment and management

*This section provides information on medicines used in CVD treatment expressed in DDD and in DDD per 1000 inhabitants per day (DID), split by the main subgroups of the ATC classification, including a narrative description of the quarter-to-quarter trends in consumption and prices over 2019-21.*

#### Antihypertensives

The consumption of antihypertensives in Georgia was the highest among the three countries, at 188 DID, which is comparable to certain OECD countries, such as Turkey (154 DID in 2019), Latvia (190 DID in 2019, 203 DID in 2020), but lower than the average across OECD countries (328 DID in 2019). The consumption of antihypertensives in Uzbekistan was almost 5 times lower than in Georgia (2020), half the level in Azerbaijan (2021), and 10 times below the average across the OECD-29 (2019)(Fig 6*)*.(1) The most consumed antihypertensives in all countries studied were ACE inhibitors and ARBs (Fig 7).

##### Figure 6.

*Consumption of antihypertensives* per 1000 people per day in 2019-2021*

** Data refer to the sum of the following ATC classes: C02 - antihypertensives, C03 - diuretics, C07 - beta blocking agents, C08 - calcium channel blockers, C09 - agents acting on the renin-angiotensin system.*

Source: OECD, 2021 (1)

##### Figure 7. Consumption of medicines used in CVDs treatment, by pharmacological subgroup, expressed in DDD per 1000 inhabitants per day, 2019-21.

CC blockers - calcium channel blockers, ACE -angiotensin converting enzyme, ARB - angiotensin II receptor blocker.

In Georgia, prices of medicines for CVD management increased by an average 45% over 2019-2021, while in Azerbaijan prices increased by 5.6% (95%CI ±11.7%) and in Uzbekistan the prices increased by an average 18% (95%CI ±3%) over 2019-20 (Fig 8).

Uzbekistan saw very low consumption of ACE inhibitors in the end of 2019 and the first half of 2020, followed by jumps in consumption and prices. Price increases occurred in most cases during Q3-Q4 2020, which coincided with the peak of COVID-19 pandemic. Moreover, in Uzbekistan, the average cost of 1 DDD for at least half of 54 common INNs for CVDs treatment was higher than in Azerbaijan and Georgia, and the average price exceeded that in Azerbaijan by a factor of 2.5 in 2019 and 2.0 in 2020, and exceeded the price in Georgia by a factor of 1.5 in 2019 and by at least 7% in 2020 (Fig 9, Appendix 3).

The consumption of medicines for CVD increased in Azerbaijan over 2019-20 across almost all subgroups of medicines except CC blockers. Consumption in 2020 increased by 10% for ACE inhibitors and by 17% for diuretics in 2020, followed by reductions through 2021 (Fig 8). Trends in prices for antihypertensives varied, ranging from a decrease of 12% for hydrochlorothiazide and potassium-sparing agents to a rise of 16.8% for propranolol in 2020. Despite price fluctuations, overall average price growth for antihypertensives was 3.5% in 2020, and retail prices were below the prices registered with the Tariff (Price) Council.(2)

##### Figure 8. Quarter-to-quarter fluctuation in prices and consumption of medicines used in CVD treatment in Azerbaijan, Georgia, and Uzbekistan, 2019-2021

*Consumption data in figures 8.1-8.6 are expressed in DDDs and average prices per SKU in mean ± 95% confidence interval (CI). The background image shows the number of COVID-19 cases in each country.*

*Figure 8.1*

*Quarter-to-quarter fluctuation in prices and consumption of medicines used in CVD treatment in Azerbaijan, 2019-2021*

Medicines used in CVD treatment totalled 90 INNs in 2019, 81 INNs in 2020, and 89 INNs in 2021. Average prices increased by 5.6% (95%CI ±11.7%) over 2019-21, which can be broken down as increases in the prices of 41 INNs (48% of available INNs) by an average of 20% (95%CI ±1.7%), and decreases in the prices of 45 INNs (53% of available INNs) by an average of 6.2% (95%CI ±1.6%). Consumption increased by 35% over 2019-21, with the biggest jump seen in Q4 2020, with a jump of 24% accompanied by growth in prices of 18%.

*Figure 8.2*

*Quarter-to-quarter fluctuation in prices and consumption of medicines used in CVD treatment in Georgia, 2019-2021*

Medicines used in CVD treatment were totalled 91 INNs in 2019, 93 INNs in 2020, and 97 INNs in 2021. Average prices increased by 40.7% (95% CI±8.1%) over 2019-21, which can be broken down as increases in price for 91 INNs (93.8% of all INNs) by an average 50.8% (95%CI ± 6.9%), and decreases in price for 6 INNs (6.2% of all INNs) by an average 42.5% (95%CI±25.5%). The most notable jumps in price werean increase of 16% in 2020 Q1 and an increase of 12% in 2021 Q1, the latter coinciding with a peak in consumption. Overall, consumption increased by 21% over 2019-21, with a quarter-on-quarter changes in consumption of +2% (95% CI±3.9%), excepting a jump of 16% in the first quarter of 2021.

*Figure 8.3*

*Quarter-to-quarter fluctuation in prices and consumption of medicines used in CVD treatment in Uzbekistan, 2019-2020*

Medicines used in CVD treatment totalled 94 INNs in 2019 and 93 INNs in 2020, while only INNs were available in both 2019 and 2020. Average price increased 17.8% (95%CI ±3%) over 2019-20, which can be broken down as increases in price for 60 INNs (89.6% of all INNs) by an average 21.2% (95%CI±5%) and decreases in price for 7 INNs (10.4% of all INNs) by an average 10.1% (95%CI±1.5%). In Q2 2020 prices jumped by 33%, which was accompanied by a 5% decrease consumption and the beginning of the COVID-19 pandemic. There were increases of 24% and 38% in consumption in 2020 Q3 and 2020 Q4, coinciding with the peak of the COVID-19 pandemic. Overall consumption of medicines for CVD treatment increased by 18% over 2019-20. High quarter-to-quarter variation was observed in consumption, with changes ranging from a rise of 55% in Q3 2019 to a decrease of 39% in Q4 2019.

##### Figure 9. Cost of 1 DDD for the most consumed medicines by ATC 2 or ATC 3 levels in Azerbaijan, Georgia, and Uzbekistan in 2019-2021 (or the nearest year)

*Figures 9.1-9.6 illustrate the average cost of 1 DDD for the most consumed medicines within each of the following ATC classes: C02 - antihypertensives, C03 - diuretics, C07 - beta blocking agents, C08 - calcium channel blockers, C09 - agents acting on the renin-angiotensin system, where ACE - angiotensin converting enzyme, ARB - angiotensin II receptor blocker.*

##### Figure 9.1. Diuretics: Cost of 1 DDD for spironolactone, US$

##### Figure 9.2. Beta blockers: Cost of 1 DDD for bisoprolol, US$

##### Figure 9.3. Calcium channel blockers: Cost of 1 DDD for amlodipine, US$

##### Figure 9.4. ACE inhibitors: Cost of 1 DDD for enalapril, US$

##### Figure 9.5. ARB: Cost of 1 DDD for losartan, US$

##### Figure 9.6. Lipid modifying agents: Cost of 1 DDD for atorvastatin, US$

#### Lipid modifiers

Lipid modifiers are used to lower cholesterol and triglyceride levels for the prevention of strokes and heart attacks. The consumption of lipid modifiers in Georgia was the highest among the three countries and amounted to 28.0 DID, which is comparable with certain OECD countries, such as Turkey in 2019,*(1)* but 5.5 times below the OECD average (113 DID in 2019). The consumption of lipid modifiers in Uzbekistan was 8.7 times lower than in Georgia (2020), 5.2 times lower than in Azerbaijan (2020), and 47 times lower than the than OECD-29 average (2019)*(*Fig 10*).(1)*

##### Figure 10. Consumption of lipid modifying agents in the retail network, DDD per 1000 people per day, 2019-2021

OECD data from OECD, 2021*(1)*

In Georgia, the consumption of lipid modifies increased by 16% in 2020 and by 35% in 2021, reaching 28.0 DID. In Azerbaijan, it increased by 12% and 13% in 2020 and 2021, reaching 14 DID. In Uzbekistan there was a reduction in the consumption of lipid modifiers by 13% over 2019-20 (Fig 11).

In Uzbekistan, consumption fluctuated markedly from quarter to quarter, with the consumption of atorvastatin falling by 26% in Q2 2019, then jumping by 69% in Q3 2019, and falling again by 36% in Q4 2019 and by 27% in Q2 2020, lastly jumping by 54% in Q3 2020. The earlier reductions in consumption during the beginning of the COVID-19 pandemic were accompanied by increases of 17% (Fig 11.3). In Azerbaijan, despite the consumption of atorvastatin fluctuating by ±10% from quarter to quarter, prices remained stable (fluctuating by ±2% quarter-to-quarter)(Fig 11.1). In Georgia, wide fluctuation in the consumption of atorvastatin (with drops of 12% in Q4 2020 and jumps of 63% in Q1 2021) was accompanied by a continuous rise in prices, with jumps of 31% in Q1 2020 and 16% in Q1 2021 (Fig 11.2). At the same time, the cost of atorvastatin in Uzbekistan was 3 times lower than in Azerbaijan and 4.6 times lower than in Georgia, in 2020 (Fig 9.6, Appendix 3).

##### Figure 11. Quarter-to-quarter fluctuation in the consumption and prices of atorvastatin in the retail network, 2019-2021

*In figures 11.1-11.3, data refer to the quarterly consumption of atorvastatin in SKUs and fluctuation in prices per SKU in 2019-2021 (or the nearest year)*

##### Figure 11.1. Azerbaijan: Quarter-to-quarter fluctuation in the consumption and prices of atorvastatin in the retail network, 2019-2021

##### Figure 11.2. Georgia: Quarter-to-quarter fluctuation in the consumption and prices of atorvastatin in the retail network, 2019-2021

##### Figure 11.3. Uzbekistan: Quarter-to-quarter fluctuation in the consumption and prices of atorvastatin in the retail network, 2019-2021

### Section 2. Medicines for diabetes management

*This section outlines medicines used in diabetes management in the outpatient setting, with consumption expressed in DDD, and in DDD per 1000 inhabitants per day (DID), with a focus on the ATC A10 subgroup - blood glucose lowering drugs (excluding insulins), illustrating quarter-to-quarter trends in consumption and prices in 2019-21.*

Overall consumption of medicines used in diabetes was highest in Georgia at 54.7 DID, which is comparable with certain OECD countries such as Lithuania, Estonia, and Latvia, but lower than OECD average of 71 DID (Fig 12).*(1)* Consumption of antidiabetic medicines in the retail networks of Azerbaijan and Uzbekistan was 14 times lower than in Uzbekistan (2020) and consumption in Azerbaijan was 5.5 times lower (2021) than the average across OECD-29 (2019).*(1)* The price of metformin in Georgia increased by 36% in Q1 2020, and by 37% in Q1 2021. In Uzbekistan, the price of metformin increased by 3% in Q3 2020, coinciding with the peak of the COVID-19 pandemic in the country. In Azerbaijan, quarter-on-quarter price fluctuation was the lowest, with price increases of 1.7% in Q3 2020 and 3.2% in Q4 2021 (Appendix 3). At the same time, the of metformin was 82% higher in Uzbekistan than in Georgia and 30% higher than in Azerbaijan in 2020. A similar comparison was seen for glimepiride, whose price was 58% higher than in Georgia and 57% higher than in Azerbaijan (Appendix 3).

##### Figure 12. Consumption of blood glucose lowering drugs in retail network, in DDD per 1000 people per day, 2019-2021.

Source for OECD consumption – OECD, 2021*.(1)*

##### Figure 13. Quarter-to-quarter fluctuation in the consumption and prices of blood sugar lowering medicines (excluding insulins), in 2019-21.

| **Azerbaijan** | **Georgia** | **Uzbekistan** |
| --- | --- | --- |
|  |  |  |

### Section 3. Medicines for asthma and COPD

*This section outlines medicines used in asthma and COPD management in outpatient settings, expressed in DDD and in DDD per 1000 inhabitants per day (DID), with a focus on the R03 category of the ATC classification, illustrating quarter-to-quarter trends in the consumption and prices in 2019-21.*

The overall consumption of bronchodilators for asthma and COPD increased by 34% in Azerbaijan in 2021, after decreasing by 10% in 2020. Azerbaijan had the highest consumption of bronchodilators, compared to Georgia and Uzbekistan (Fig 14), albeit with wide fluctuations in the consumption of bronchodilators, with drops of 70% in Q2 2020 and Q1 2021, and price increases of over 60% in Q3 2020 and Q2 2021 (Fig 15). The consumption of bronchodilators in Uzbekistan was at least 5 times lower than in Azerbaijan and 3.5 times lower than in Georgia in 2020 (3.51 DID in 2019 and 4.94 DID in 2020). In Georgia, consumption fluctuated widely, with an overall decrease in consumption of 35% from 2019 Q2 to 2020 Q2. Decreases in consumption were accompanied with prices declining in Q1 2021 and coincided with the peak of the COVID-19 pandemic (Appendix 3).

The lowest levels of price fluctuation were seen in Azerbaijan (quarter-on-quarter variability of <5%), while in Georgia prices for bronchodilators jumped by 35% in 2020 and fell by 29% in 2021 (Fig 15). The highest prices of budesonide and montelukast were seen in Georgia. The highest prices of salbutamol were seen in Uzbekistan, where the price exceeded by 2.5 times the average price in Georgia and exceeded by 80% the price in Azerbaijan in 2020 (Appendix 3).

##### Figure 14. Consumption of bronchodilators in the retail network, in DDD per 1000 people per day, 2019-2021.

##### Figure 15. Quarter-to-quarter fluctuation in the consumption and prices of bronchodilators, in 2019-21.

| **Azerbaijan** | **Georgia** | **Uzbekistan** |
| --- | --- | --- |
|  |  |  |

Data reflect the total consumption of medicines included in ATC group R03.

### Section 4. Medicines for cancer management

*This section outlines medicines used for endocrine therapy in breast and prostate cancer, in the outpatient setting, with a focus on ATC group L02, illustrating quarter-to-quarter trends in the consumption and prices in 2019-21.*

Consumption of medicines for cancer treatment (outpatient endocrine therapy for breast and prostate cancer) in the retail network increased in Azerbaijan (by 7% in 2020, by 54% in 2021) and in Georgia (by 21% in 2020, by 16% in 2021), while in Uzbekistan consumption decreased by a factor of 3 over 2019-20. Georgia had the highest consumption, compared to Azerbaijan and Uzbekistan (Fig 16). In Azerbaijan, despite little quarter-to-quarter change in prices, prices for almost all subgroups of medicines for endocrine therapy in cancer were the highest across the three countries (Appendix 3). In Georgia, an increase in consumption was accompanied by decreases in prices (Fig 17). In Uzbekistan, low levels of consumption, possibly reflecting shortages and/or uneven supply, were accompanied by price increases during the peak of the COVID-19 pandemic (Fig 17, Appendix 3).

##### Figure 16. Consumption of endocrine therapies for breast and prostate cancer in the retail network, in DDD per 1000 people per day, 2019-2021.

##### Figure 17. Quarter-to-quarter fluctuation in the consumption and prices of medicines used in hormone therapy in breast and prostate cancer, 2019-21.

| Azerbaijan | Georgia | Uzbekistan |
| --- | --- | --- |
|  |  |  |

### Data reflect the total consumption of medicines included in ATC group L02.

### Section 5. Medicines for epilepsy management

*This section outlines medicines used in epilepsy in the outpatient setting, focusing on the N03 category of the ATC classification, illustrating quarter-to-quarter changes in consumption and prices in 2019-21.*

Epilepsy is one of the most common neurological diseases, affecting around 49.5 million people of all ages around the world.(4) Up to 70% of people with epilepsy could live seizure-free if appropriately treated with antiseizure medicines, but there is an estimated treatment gap of 40% in the European region.(5) The high global burden of epilepsy requires prevention where possible.

First-line antiepileptic drugs (AEDs; such as phenobarbital, phenytoin, carbamazepine, and valproic acid) are a highly cost-effective use of health resources.(6) All first-line medicines are included in the WHO Model List of Essential Medicines.(7)

In Uzbekistan, the consumption of AEDs decreased by 16% (from 1.51 in 2019 to 1.28 DID in 2020), and consumption was the lowest across the three countries (Fig 18), while the standardized death rate from epilepsy was the highest (2.40/100,000 inhabitants in 2020). In Georgia, there was high consumption of AEDs, although during the COVID-19 pandemic the consumption of AEDs decreased by 38% in 2020 (from 4.38 to 2.70 DID), followed by an increase of 26% in 2021, up to 3.40 DID. Despite the reduction in consumption of AEDs over 2019-21 in Georgia, the consumption level remained at least 2-fold higher than in Azerbaijan in 2021 and Uzbekistan in 2020 (Fig 19). The highest consumption was seen for the first-generation AEDs (83% of total AED consumption in 2021). In Georgia, prices increased by 39% for carbamazepine and by 44% for valproate over 2019-21, especially in Q3 2020 (a price jump of 14.6% for carbamazepine) and Q4 2020 (a price jump of 12.8% for valproate), coinciding with the peak of the COVID-19 pandemic in the country (Appendix 3).

In Azerbaijan, there was moderate quarter-to-quarter fluctuation in consumption and little fluctuation in prices: the price of valproate decreased by 10% over 2019-21 while the price of carbamazepine increased by 1%. However, the cost of AEDs in Azerbaijan was higher than in Georgia: the price of carbamazepine was higher by 27% in 2020 and by 18% in 2021, and the price valproate was higher by 35% in 2020 and by 27% in 2021. Compared with Uzbekistan, the cost of valproate in Azerbaijan was 27% lower and for carbamazepine was 33% higher, in 2020 (Appendix 3).

The market share for second-generation AEDs increased from 16% to 17% over 2019-21 in Georgia, and from 22% to 29% in Azerbaijan, while in Uzbekistan it reduced from 25% in 2019 to 18% in 2020 along with very low consumption overall, possibly indicating shortages, in 2020-21. The most consumed second-generation AEDs were different in each country. In Azerbaijan, the most consumed in 2021 were levetiracetam (12.1% of consumption) and pregabalin (8%), while in Georgia the most consumed were lamotrigine (8.7%) and levetiracetam (7.6%). In Uzbekistan, the market share of the second generation of AEDs decreased from 25.2% in 2019 to 18.0% in 2020, while the most consumed second-generation AEDs were pregabalin in 2019 (15.2%) and gabapentin in 2020 (12%). In all three countries, consumption of second- and third-generation AEDs was very low, possibly indicating shortages (Appendix 3).

##### Figure 18. Consumption of antiepileptic drugs in the retail network, in DDD per 1000 people per day, 2019-2021.

##### Figure 19. Quarter-to-quarter changes in the consumption and prices of medicines used in epilepsy management, 2019-21.

| Azerbaijan | Georgia | Uzbekistan |
| --- | --- | --- |
|  |  |  |

### Data reflect the total consumption of medicines included in ATC group N03.

### Section 6. Medicines for mental health disorders

*This section outlines medicines used in anxiety and depressive disorders in the outpatient setting, expressed in DDD and DDD per 1000 inhabitants per day (DID), focusing on the N03 category of the ATC classification, illustrating quarter-to-quarter changes in consumption and prices in 2019-21.*

Mental disorders are a growing concern in the 21st century. The most common mental health disorders include depression and anxiety, with a prevalence estimated at around 4 percent of the global population.(8) The COVID-19 pandemic triggered a 25% increase in prevalence of anxiety and depression worldwide.(8,9) Since the start of the COVID‑19 crisis, the consumption of medicines for the treatment of anxiety and depression has increased by 25% in Azerbaijan (from 5.95 to 7.33 DID) and by 7.7% in Georgia (from 14.24 to 15.34 DID) over 2019-21, although there was a temporary decrease in consumption in 2020 in Georgia, with Uzbekistan also having decreased consumption in 2020 (Fig 20). Uzbekistan had the lowest level of consumption across the three countries, accompanied by possible interruptions in supply in Q4 2019–Q2 2020 (Appendix 3).

Despite the increase in consumption of anxiolytics and antidepressants in 2019-2021, the consumption of antidepressants in Georgia (8.61 DID on average) was 7.7 times lower than the average across OECD countries (66 DID) in 2019 and 2.7 times lower than in Latvia (18 DID), while the consumption of antidepressants was lower in Azerbaijan and Uzbekistan compared to Georgia in 2021, by 24% and a factor of 8, respectively (Fig 21).(1)

##### Figure 20. Consumption of medicines for anxiety and depression in the retail network, DDD per 1000 people per day, 2019-2021*

##### Figure 21. Consumption of antidepressants in Azerbaijan, Georgia, and Uzbekistan with OECD countries, in DDD per 1000 people per day in 2019-2021.

##### Figure 22.

*Quarter-to-quarter fluctuation in the consumption and prices of medicines used in anxiety and depressive disorders treatment in retail network in outpatient settings, 2019-21*

| Azerbaijan | Georgia | Uzbekistan |
| --- | --- | --- |
|  |  |  |

Data reflect the total consumption of medicines included in ATC groups N05B and N06A.

**References**

1. Pharmaceutical consumption. In: Health at a Glance 2021 [Internet]. OECD; 2021 [cited 2022 Nov 30]. (Health at a Glance). Available from: https://www.oecd-ilibrary.org/social-issues-migration-health/health-at-a-glance-2021_5689c05c-en

2. Tariff (price) Council of the Republic of Azerbaijan. Prices on state-registered pharmaceuticals [Internet]. 2017. Available from: http://www.tariffcouncil.gov.az/documents/DVM-530.pdf

3. WHO Collaborating Centre for Drug Statistics. ATC/DDD Index 2022 [Internet]. 2022. Available from: https://www.whocc.no/atc_ddd_index/

4. Knezevic CE, Marzinke MA. Clinical Use and Monitoring of Antiepileptic Drugs. The Journal of Applied Laboratory Medicine. 2018 Jul 1;3(1):115–27.

5. De Boer HM. “Out of the Shadows”: A Global Campaign Against Epilepsy. Epilepsia. 2002 Oct 24;43:7–8.

6. World Health Organization. Epilepsy: a public health imperative [Internet]. 2019. Available from: https://apps.who.int/iris/handle/10665/325293

7. World Health Organization. WHO model list of essential medicines - 22nd list, 2021 [Internet]. 2021 [cited 2022 Oct 6]. Available from: https://www.who.int/publications/i/item/WHO-MHP-HPS-EML-2021.02

8. WHO Regional Office for Europe. Prevention and management of mental health conditions in Uzbekistan: the case for investment. 2021.

9. World Health Organization. Scientific brief: COVID-19 pandemic triggers 25% increase in prevalence of anxiety and depression worldwide [Internet]. 2022. Available from: https://www.who.int/news/item/02-03-2022-covid-19-pandemic-triggers-25-increase-in-prevalence-of-anxiety-and-depression-worldwide
