# Supplementary material for: Trends in cost and consumption of essential medicines for non-communicable diseases in Azerbaijan, Georgia, and Uzbekistan, from 2019 to 2021
Source: PLoS One. 2023 Dec 7;18(12):e0294680. doi: 10.1371/journal.pone.0294680 (PMC10703197; doi:10.1371/journal.pone.0294680)
Supplement: S3 File — (DOCX) [file pone.0294680.s003.docx]

## Appendix 3. Comparison of the consumption and prices of NCD medicines in Azerbaijan, Georgia, and Uzbekistan in 2019-2021, analysis by disease group.

### Section 1: Cardiovascular disease

This section provides information on CVD medicines, analysed overall and by therapeutic subgroups.

Table 1. Comparison of key parameters in the consumption and pricing of medicines for CVD treatment and management in Azerbaijan, Georgia, and Uzbekistan

| **Azerbaijan** | **Georgia** | **Uzbekistan** |
| --- | --- | --- |
| ***Medicines for CVDs treatment and management in outpatient settings*** | | |
| Consumption of antihypertensives^1^ was low to moderate: average daily consumption per capita increased by 9% in 2020 (from 61 to 67 DID) and 5% (up to 70 DID) in 2021 compared to the previous year (table 2).  Quarter-to-quarter variation in average price was low (<5% on average).  However, quarter-to-quarter variation in consumption was significant. For bisoprolol (representing nearly 45% of all beta blockers consumed), consumption fell by 34% in Q2 2020 followed by a jump of 42% in Q3 2020. Consumption of amlodipine (over 75% of the calcium channel blocker consumption) fell by 17% in Q2 2020 but jumped by 24% in Q4 2020. Consumption of captopril (over 49% of the ACE inhibitor consumption) well by 28% in Q2 2020, jumped by 23% in Q4 2020, fell again by 14% in Q2 2021 and jumped by 36% in Q4 2021.  Prices of key CVD medicines in Azerbaijan were often at a level between the prices seen in Georgia and Uzbekistan. The price of bisoprolol was 2.7 folds lower than in Uzbekistan (2019-2020), but 13% higher than in Georgia (2021). The price of amlodipine was 34%-36% lower than in Georgia and 1.6 times below that in Uzbekistan. The price of enalapril was higher than in Georgia – by 37% in 2020 and 24% in 2021 – but 9 times lower than in Uzbekistan (in 2020) (table 2, Fig.2). The price of spironolactone was higher than in Georgia (by 12% in 2020, by 5% in 2021) and Uzbekistan (by 38% in 2020). The price of losartan was lower than in Georgia (by 50% in 2020, by 70% in 2021) and 3 folds higher than in Uzbekistan in 2020. The price of atorvastatin was lower than in Georgia (by 34% in 2020 and by 27% in 2021), but 3 times the price in Uzbekistan (Fig.2, table 2). | Consumption levels for antihypertensives^1^ were moderate but increased by 6% in 2020 (from 160 to 170 DID) and 11% (to 188 DID) in 2021, compared to previous years. These levels are about half the average among OECD countries (328 DID in 2019, but comparable to levels in Turkey (154 DID) and Latvia (190 DID)(table 2).  There was some fluctuation in the consumption of medicines for CVDs, but consumption gradually increased over 2019-21, and was seemingly unrelated to peaks in the COVID-19 pandemic. Large quarter-to-quarter consumption fluctuations were seen for amlodipine (representing over 60% of calcium channel blocker consumption), for which consumption jumped by 35% the first quarter of 2021, coinciding with the peak of the COVID-19 pandemic and, followed by a drop of 24% in the third quarter of 2021; bisoprolol (representing 35% of beta blocker consumption), which saw consumption rise by 23% in Q4 2019, by 14% in Q3 2020, and by 20% in Q1 2021; enalapril in combination with diuretics (representing almost 25% of the total consumption of ACE inhibitors and ARBs), which saw variability in consumption of ±16% in 2019-2021; losartan, which saw a 50% fall in consumption in Q2 2019, jumped by 37% in Q4 2019, by 33% in Q1 2021, and fell by 8-10% in Q3 and Q4 2021.  Large price increases were seen for almost all subgroups of antihypertensives in 2020, decelerating in 2021. Thus, the average price growth for diuretics was 23.5% in 2020 and 7.5% in 2021, for beta blockers 27.5% in 2020, 29.6% in 2021, for calcium channel blockers 7.5% in 2020, and 18% in 2021 18%, for ACE inhibitors combinations 14.1% in 2020 and 7.6% in 2021, for ARBs 56.2% in 2020 and 4.2% in 2021, for ARB combinations 20.9% in 2020 and 6.3% in 2021. However, decreasing prices (mean decrease 8%±0.099, median 5%) were observed for 30% of INNs (25 out of 83) available in the Georgian pharmaceutical retail network in 2021 for CVD treatment and management.  The average cost of 1 DDD for antihypertensives was higher than in Azerbaijan by 24.6% in 2020 and 33.4% in 2021, and lower than in Uzbekistan by 63% in 2019 and 44% in 2020 , however price comparisons within the group were multidirectional, for example the cost of 1 DDD for captopril was higher than in Azerbaijan (by 15% in 2020, by 28% in 2021) but lower than in Uzbekistan (by 13% in 2019 by 19% in 2020), while for enalapril the price in Georgia was lower than in Azerbaijan by 27% in 2020 and by 19% in 2021, and 13 times lower than in Uzbekistan in 2020. | Consumption levels for antihypertensives were very low (32 DID in 2019 and 35 DID in 2020), at least 2 times lower than in Azerbaijan and 5 times lower than in Georgia, in 2020.  There was a high level of fluctuation in the consumption of medicines for CVD treatment and management. The consumption of bisoprolol (representing 20% of beta blocker consumption) jumped by a factor of 2.3 in the beginning of the COVID-19 pandemic, followed by a reduction of 40% in Q2-Q3 2020 and another jump of 69% in Q4 2020; a similar pattern was seen for the consumption of amlodipine (representing over 65% of calcium channel blocker consumption), which increased by a factor go 2.7 folds in Q3 2019, by 77% in Q2 2020, and by 46% in Q3 2020, followed by decreases of 75% in Q4 2020 and 22% in Q4 2020. The consumption of losartan (representing over 30% of ACE inhibitor and ARB consumption) showed dips of 41% in Q2 2019, 56% in Q4 2019, and 57% in Q4 2020, and jumps of 2.3 folds in Q3 2019, 27% in Q1 2020, and 36% in Q3 2020 (table 2, Fig 1).  In Uzbekistan, 60 INNs were available for CVDs treatment in 2019-20, out of which prices of 60 INNs (89.6%) increased by 21.2% (CI±5%). In 2020, price increases occurred in Q3-Q4 2020 in more than half of cases, which coincided with the peak of the COVID-19 pandemic in Uzbekistan and the introduction of a new price regulation system, and may reflect irregular supply of medicines prior to the pandemic (i.e. in 2019) and in the beginning of the COVID-19 pandemic.  The average cost of 1 DDD for CVD treatment in 2019-2020 was higher than in Azerbaijan (by a factor of 2.7 in 2019,by a factor of 2.2 in 2020) and Georgia (by a factor of 2.7 in 2019, by 80% in 2021). As illustrative examples, the cost of 1 DDD of bisoprolol (a beta blocker) was $0.52 and $0.55 in 2019 and 2020, which is 2.7 times higher than in Azerbaijan, while the cost of 1 DDD for metoprolol (representing 25% of the consumption of beta blockers in 2020) was 20 times lower than in Georgia and 6 times lower than in Azerbaijan, in 2020. At the same time, the cost of 1 DDD of amlodipine was comparable in Uzbekistan ($0.037) and Azerbaijan ($0.041) and 32% lower than in Georgia in 2019, but increased to reach a price 6% above that in Georgia (Fig.2).  The cost of 1 DDD for enalapril (3.7% of ACE inhibitors and ARB consumption) was 9 times higher than in Azerbaijan and 13 times higher than in Georgia, in 2020. At the same time, the cost of 1 DDD for losartan (over 20% of the ACE inhibitor and ARB consumption) was 8% higher than in Azerbaijan but 46% lower than in Georgia, in 2020 (Fig.5).  The cost of 1 DDD of spironolactone (representing 25% of diuretic consumption in 2020) was 27% lower than in Azerbaijan and 18% lower than in Georgia, in 2020 (Fig.2). |
| Figure 1. Consumption and prices for enalapril in Azerbaijan, Georgia, and Uzbekistan, 2019-2021. | | |
|  |  |  |
|  |  |  |
| Figure 2. Cost of 1 DDD for CVD medicines in Azerbaijan, Georgia, and Uzbekistan, 2019-2021, US$ | | |
|  |  |  |
|  |  |  |

ACE - angiotensin converting enzyme, ARB - angiotensin II receptor blocker.

Table 2. Trends in the consumption of medicines for CVD treatment and management in Azerbaijan, Georgia, and Uzbekistan.

| **Medicine** | **Indicator** | **Azerbaijan** | | | **Georgia** | | | **Uzbekistan** | |
| --- | --- | --- | --- | --- | --- | --- | --- | --- | --- |
|  |  | 2019 | 2020 | 2021 | 2019 | 2020 | 2021 | 2019 | 2020 |
| Antihypertensives^1^ | Consumption in DID | 61.34 | 66.81 | 70.17 | 160.10 | 169.91 | 188.19 | 31.68 | 34.60 |
|  | Number of different products available | 393 | 341 | 372 | 516 | 497 | 495 | 384 | 420 |
| С03 diuretics | Consumption in DID | 4.29 | 5.00 | 4.64 | 17.20 | 14.71 | 16.06 | 2.08 | 3.25 |
|  | Number of different products available | 40 | 35 | 37 | 53 | 53 | 53 | 34 | 40 |
| C03DA01 spironolactone | Cost of 1 DDD, US$ [mean (± 95%CI)] | $0.25 (CI±0.002) | $0.25 (CI±0.004) | $0.24 (CI±0.001) | $0.18 (CI±0.006) | $0.22 (CI±0.006) | $0.23 (CI±0.008) | $0.18 (CI±0.013) | $0.18 (CI±0.004) |
|  | Consumption in DID | 0.75 | 0.86 | 0.85 | 2.10 | 2.06 | 2.73 | 0.59 | 1.08 |
|  | Number of different products available | 11 | 11 | 11 | 7 | 6 | 7 | 5 | 9 |
|  | Proportion of overall C03 consumption | 36.0% | 38.4% | 35.9% | 36.4% | 42.5% | 48.8% | 28% | 25% |
| C07 beta blockers | Consumption in DID | 9.61 | 10.68 | 11.02 | 18.55 | 19.02 | 21.57 | 3.36 | 4.04 |
|  | Number of different products available | 77 | 61 | 69 | 118 | 109 | 107 | 68 | 79 |
| C07AB07 bisoprolol | Cost of 1 DDD, US$ [mean (± 95%CI)] | $0.19 (CI±0.002) | $0.20 (CI±0.002) | $0.21 (CI±0.002) | $0.16 (CI±0.001) | $0.19 (CI±0.001) | $0.19 (CI±0.003) | $0.52 (CI±0.047) | $0.55 (CI±0.085) |
|  | Consumption in DID | 3.86 | 4.45 | 4.55 | 5.22 | 6.98 | 8.49 | 1.04 | 1.17 |
|  | Number of different products available | 18 | 12 | 14 | 33 | 27 | 30 | 33 | 47 |
|  | Proportion of overall C07 consumption | 42.1% | 44.3% | 44.6% | 24.8% | 31.0% | 34.5% | 20% | 20% |
| C07AB02 metoprolol | Cost of 1 DDD, US$ [mean (± 95%CI)] | $0.10 (CI±0.001) | $0.11 (CI±0.001) | $0.10 (CI±0.001) | $0.28 (CI±0.005) | $0.36 (CI±0.004) | $0.42 (CI±0.008) | $0.005 (CI±0.001) | $0.01 (CI±0.003) |
|  | Consumption in DID | 1.08 | 1.15 | 1.19 | 3.08 | 2.92 | 3.32 | 1.00 | 0.80 |
|  | Number of different products available | 14 | 10 | 9 | 17 | 16 | 18 | 5 | 5 |
|  | Proportion of overall C07 consumption | 15.9% | 15.2% | 14.7% | 31.6% | 28.5% | 29.4% | 35% | 25% |
| С08 calcium channel blockers (CCB) | Consumption in DID | 7.05 | 6.90 | 7.00 | 18.70 | 18.69 | 20.26 | 6.01 | 4.55 |
|  | Number of different products available | 46 | 32 | 39 | 58 | 52 | 53 | 55 | 46 |
| C08CA01 amlodipine | Cost of 1 DDD, US$ [mean (± 95%CI)] | $0.04 (CI±0.001) | $0.04 (CI±0.001) | $0.03 (CI±0.001) | $0.05 (CI±0.001) | $0.06 (CI±0.001) | $0.06 (CI±0.001) | $0.03 (CI±0.005) | $0.06 (CI±0.001) |
|  | Consumption in DID | 6.17 | 6.16 | 6.37 | 12.39 | 12.73 | 14.28 | 5.15 | 3.89 |
|  | Number of different products available | 28 | 22 | 26 | 33 | 30 | 34 | 35 | 34 |
|  | Proportion of overall C08 consumption | 71.6% | 75.9% | 78.1% | 59.2% | 60.6% | 65.7% | 68% | 66% |
| С09 agents acting on the renin-angiotensin system | Consumption in DID | 39.39 | 43.22 | 46.44 | 95.18 | 108.42 | 120.31 | 19.27 | 21.51 |
|  | Number of different products available | 217 | 200 | 215 | 268 | 261 | 261 | 213 | 242 |
| ACE inhibitors (alone and in combination with other medicines) | Consumption in DID | 30.48 | 33.28 | 34.45 | 81.02 | 86.94 | 91.30 | 7.18 | 9.19 |
|  | Number of different products available | 133 | 122 | 129 | 174 | 170 | 165 | 98 | 118 |
| C09AA01 captopril | Cost of 1 DDD, US$ [mean (± 95%CI)] | $0.07 (CI±0.001) | $0.07 (CI±0.001) | $0.07 (CI±0.001) | $0.07 (CI±0.001) | $0.08 (CI±0.002) | $0.09 (CI±0.001) | $0.08 (CI±0.015) | $0.10 (CI±0.199) |
|  | Consumption in DID | 4.24 | 4.46 | 4.65 | 12.69 | 14.54 | 15.28 | 0.07 | 0.05 |
|  | Number of different products available | 11 | 9 | 11 | 31 | 28 | 28 | 2 | 2 |
|  | Proportion of overall C09 consumption | 15.9% | 15.1% | 14.8% | 14.7% | 15.9% | 14.6% | 0.2% | 0.1% |
| C09AA02 enalapril | Cost of 1 DDD, US$ [mean (± 95%CI)] | $0.06 (CI±0.001) | $0.06 (CI±0.001) | $0.06 (CI±0.001) | $0.03 (CI±0.006) | $0.04 (CI±0.002) | $0.04 (CI±0.001) | $0.91 (CI±0.106) | $0.56 (CI±0.27) |
|  | Consumption in DID | 3.85 | 3.85 | 3.64 | 4.84 | 5.65 | 6.14 | 0.67 | 0.89 |
|  | Number of different products available | 18 | 17 | 16 | 14 | 14 | 15 | 22 | 26 |
|  | Proportion of overall C09 consumption | 13.0% | 12.3% | 10.7% | 5.3% | 5.0% | 4.9% | 3.6% | 3.7% |
|  |  |  |  |  | *Combinations of enalapril and diuretics had the highest consumption, over 20%* | | |  |  |
| ARBs (alone and in combination with other medicines) | Consumption in DID | 8.91 | 9.94 | 11.99 | 14.16 | 21.47 | 29.01 | 12.08 | 12.32 |
|  | Number of different products available | 84 | 78 | 86 | 94 | 91 | 115 | 124 | 115 |
| C09CA01 losartan | Cost of 1 DDD, US$ [mean (± 95%CI)] | $0.10 (CI±0.002) | $0.09 (CI±0.001) | $0.09 (CI±0.001) | $0.14 (CI±0.004) | $0.19 (CI±0.009) | $0.31 (CI±0.026) | $0.08 (CI±0.012) | $0.10 (CI±0.019) |
|  | Consumption in DID | 0.76 | 0.92 | 1.00 | 1.14 | 1.34 | 1.30 | 3.94 | 3.45 |
|  | Number of different products available | 10 | 8 | 8 | 12 | 8 | 8 | 20 | 19 |
|  | Proportion of overall C09 consumption | 2.0% | 2.1% | 2.1% | 1.5% | 1.9% | 2.2% | 27.6% | 20.3% |
| C10 lipid-modifying medicines | Consumption in DID | 10.99 | 12.32 | 13.90 | 17.87 | 20.70 | 28.02 | 2.74 | 2.39 |
|  | Number of different products available | 86 | 78 | 83 | 112 | 117 | 118 | 89 | 86 |
| C10AA05 atorvastatin | Cost of 1 DDD, US$ [mean (± 95%CI)] | $0.10 (CI±0.001) | $0.10 (CI±0.001) | $0.10 (CI±0.001) | $0.15 (CI±0.002) | $0.16 (CI±0.002) | $0.14 (CI±0.002) | $0.03 (CI±0.001) | $0.03 (CI±0.001) |
|  | Consumption in DID | 4.13 | 4.15 | 4.06 | 6.33 | 7.87 | 12.07 | 1.45 | 1.16 |
|  | Number of different products available | 26 | 25 | 25 | 32 | 36 | 34 | 43 | 42 |
|  | Proportion of overall C10 consumption | 48.2% | 45.3% | 40.6% | 39.4% | 45.8% | 51.9% | 59.5% | 58.9% |

DDD - defined daily dose, CI - confidence interval, CCB - calcium channel blockers, ACE- angiotensin converting enzyme, ARB - angiotensin II receptor blockers.
^1^ In this analysis, the category ‘antihypertensives’ comprises the following ATC groups following classes: C02 - antihypertensives, C03 - diuretics, C07 - beta blocking agents, C08 - calcium channel blockers, C09 - agents acting on the renin-angiotensin system.

### Section 2: Medicines for diabetes management

This section outlines trends for medicines for diabetes management under the A10 category of ATC classification and details on the most consumed medicines within this category.

Table 3. Trends in the consumption and prices of glucose lowering medicines

| **Health condition** | **Medicine** | **Indicator** | **Azerbaijan** | | | **Georgia** | | | **Uzbekistan** | |
| --- | --- | --- | --- | --- | --- | --- | --- | --- | --- | --- |
|  |  |  | 2019 | 2020 | 2021 | 2019 | 2020 | 2021 | 2019 | 2020 |
| Diabetes | Blood glucose lowering drugs (A10) | Consumption in DID | 10.56 | 11.96 | 12.93 | 45.91 | 44.07 | 55.19 | 8.01 | 6.02 |
|  |  | Number of different products available | 66 | 53 | 57 | 80 | 80 | 74 | 115 | 120 |
|  | A10BA02 metformin | Cost of 1 DDD, US$ [mean (± 95%CI)] | $0.14 (CI±0.001) | $0.14 (CI±0.002) | $0.14 (CI±0.001) | $0.07 (CI±0.001) | $0.10 (CI±0.001) | $0.11 (CI±0.002) | $0.18 (CI±0.006) | $0.18 (CI±0.002) |
|  |  | Consumption in DID | 3.19 | 3.34 | 3.71 | 12.4 | 12.0 | 15.9 | 2.1 | 1.9 |
|  |  | Number of different products available | 21 | 18 | 19 | 29 | 30 | 28 | 39 | 41 |
|  | A10BB12 glimepiride | Cost of 1 DDD, US$ [mean (± 95%CI)] | $0.06 (CI±0.001) | $0.06 (CI±0.001) | $0.06 (CI±0.001) | $0.05 (CI±0.001) | $0.06 (CI±0.001) | $0.07 (CI±0.004) | $0.11 (CI±0.001) | $0.10 (CI±0.001) |
|  |  | Consumption in DID | 2.15 | 2.54 | 3.04 | 7.32 | 6.41 | 8.08 | 1.93 | 2.19 |
|  |  | Number of different products available | 16 | 9 | 10 | 17 | 16 | 2 | 28 | 31 |

CI - confidence interval, DID – DDD consumed per 1000 population per day.

Table 5. Comparison of key parameters in the consumption of anti-diabetic medicines in Azerbaijan, Georgia, and Uzbekistan.

| **Azerbaijan** | **Georgia** | | | **Uzbekistan** |
| --- | --- | --- | --- | --- |
| ***Medicines for diabetes management in outpatient settings*** | | | | |
| Consumption of antidiabetic medicines was low in Azerbaijan.  Quarter-to-quarter variability was low (<5%).  Decreased consumption from 2020 Q4 to 2021 Q1, coinciding with the peak of the COVID-19 pandemic.  The cost of 1 DDD of human insulin was the highest across the three countries, exceeding by a factor of 40 the cost in Georgia and by a factor of 70 the cost in Uzbekistan.  Costs for 1 DDD for glimepiride and metformin were moderate. | | Consumption of antidiabetic medicines was moderate in Georgia, comparable with some OECD countries (Lithuania, Austria, Latvia).  High quarter-to-quarter variability in prices: increases of up to 37% in Q1 2020 and Q1 2021.  Consumption was highly variable from quarter to quarter.  The cost of 1 DDD of fast-acting human insulin was the lowest across the three countries.  The costs of metformin and glimepiride were the lowest among the three countries. | Consumption of antidiabetic medicines was very low in Uzbekistan.  Quarter-to-quarter price variability was moderate (±10%).  There was an increase in consumption in Q3 2020, which coincided with the peak of the COVID-19 pandemic.  The cost of 1 DDD of insulin was moderate, the cost of 1 DDD of fast-acting human insulin was double the average cost in Georgia.  The costs of metformin and glimepiride were higher than in Azerbaijan and Georgia. | |
| Figure 3. Consumption and price of metformin in Azerbaijan, Georgia, and Uzbekistan in 2019-2021 . | | | | |
|  | |  |  | |
| Figure 4. Cost of 1 DDD for blood glucose lowering agents in 3 selected countries, 2019-2021 or the latest available year, US$ | | | | |
|  | |  |  | |

DDD – defined daily dose, SKU – stock-keeping units.

### Section 3: Medicines for asthma and COPD

This section outlines medicines used in asthma and COPD management under the R03 category of ATC classification, divided into short acting beta-2 agonists (SABA), long-acting beta2-agonists (LABA), leukotriene receptor antagonists (LTRA), and inhaled corticosteroids (ICS).

Table 6. Comparison of trends in the consumption and pricing of medicines for asthma and COPD in Azerbaijan, Georgia, and Uzbekistan

| **Azerbaijan** | **Georgia** | **Uzbekistan** |
| --- | --- | --- |
| ***Medicines for asthma and COPD in outpatient settings*** | | |
| Age-standardized deaths from asthma: 5.34 per 100,000 in 2019. Age-standardized deaths from COPD: 24.95 per 100,000 in 2019.(1) | Age-standardized deaths from asthma: 0.91 per 100,000 in 2019. Age-standardized deaths from asthma: 40.49 per 100,000 in 2019.(1) | Age-standardized deaths from asthma: 7.22 per 100,000 in 2019. Age-standardized deaths from asthma: 18.91 per 100,000 in 2019.(1) |
| Consumption of medicines for asthma and COPD was moderate, with an overall declining trend. Consumption declined by 10% in 2020 and by 34% in 2021 compared to previous years (table 8).  Quarter-to-quarter price variability was moderate (±17%).  The consumption of salbutamol fluctuated markedly, with reductions of 36% in Q2 2020 and 21% in Q3 2020, followed by an increase of 48% in Q4 2020.  Costs for 1 DDD for SABA, LABA, ICSs, and LTRA were moderate compared to the other two countries (Fig.6, table 7) | Consumption of medicines for asthma and COPD was moderate, with an overall declining trend. Consumption delinked by 17% in 2020 and by 16% in 2021 compared to previous years (table 8).  Price variability was high: +35% in 2020, -29% in 2021.  The consumption of asthma and COPD medicines fluctuated markedly, with a gradual reduction from Q3 2019 to Q2 2020, plateauing at an overall decrease in consumption of 35% in Q2 2020 compared to the same quarter in 2019. Decreases in consumption were accompanied with price decreases in Q1 2021 and coincided with the peak of the COVID-19 pandemic.  The cost of budesonide (ICS) and montelukast (LTRA) were the highest compared to the other two countries, while the cost of salbutamol was the lowest in 2019-20 and decreased further in 2021, by 80% (Fig.6, table 7). | Consumption of medicines for asthma and COPD was very low (3.51 DID in 2019 and 4.94 DID in 2020), which is at least 5 times lower than in Azerbaijan and 3.5 times lower than in Georgia in 2020.  Price variability was moderate (±10%) with jumps of up to 20%, which coincided with increases in consumption (+73%) and the peak of the COVID-19 pandemic.  The cost of salbutamol was the highest compared to the other two countries, with the price of salbutamol 2.5 times the average cost in Georgia and 80% above the cost in Azerbaijan, in 2020 (table 5).  However, the costs of budesonide, fluticasone, and montelukast were lower than in the other two countries (Fig.6, Table 7). |
| Figure 5. Consumption and price of salbutamol in Azerbaijan, Georgia, and Uzbekistan in 2019-2021 . | | |
|  |  |  |
| Figure 6. Cost of 1 DDD for bronchodilators in Azerbaijan, Georgia, and Uzbekistan, 2019-2021, US$ | | |
|  |  |  |

Table 7. Trends in the consumption and prices of medicines for asthma and COPD in Azerbaijan, Georgia, and Uzbekistan, 2019-2021.

| **Health condition** | **Medicine** | **Indicator** | **Azerbaijan** | | | **Georgia** | | | **Uzbekistan** | |
| --- | --- | --- | --- | --- | --- | --- | --- | --- | --- | --- |
|  |  |  | 2019 | 2020 | 2021 | 2019 | 2020 | 2021 | 2019 | 2020 |
| Asthma and COPD | Bronchodilators^1^ | Consumption in DID | 28.22 | 25.35 | 33.86 | 21.29 | 17.75 | 14.97 | 3.51 | 4.94 |
|  |  | Number of different products available | 123 | 118 | 131 | 132 | 139 | 128 | 112 | 127 |
|  | R03AC02  salbutamol  (SABA) | Cost of 1 DDD, US$ [mean (± 95%CI)] | $0.03 (CI±0.006) | $0.05 (CI±0.004) | $0.06 (CI±0.003) | $0.04 (CI±0.001) | $0.03 (CI±0.002) | $0.07 (CI±0.003) | $0.09 (CI±0.021) | $0.10 (CI±0.015) |
|  |  | Consumption in DID | 9.24 | 6.67 | 5.27 | 9.15 | 8.96 | 3.54 | 1.78 | 1.78 |
|  |  | Number of different products available | 9 | 9 | 9 | 18 | 17 | 19 | 9 | 9 |
|  |  | Proportion of overall R03 consumption | 31.7% | 35.1% | 27.8% | 24.2% | 24.3% | 19.5% | 43.8% | 34.8% |
|  | R03AC04  fenoterol  (SABA) | Cost of 1 DDD, US$ [mean (± 95%CI)] | $0.16 (CI±0.002) | $0.13 (CI±0.007) | $0.13 (CI±0.001) | $0.11 (CI±0.018) | $0.11 (CI±0.009) | $0.09 (CI±0.004) | $0.22 (CI±0.022) | $0.18 (CI±0.014) |
|  |  | Consumption in DID | 0.099 | 0.020 | 0.010 | 0.01 | 0.006 | 0.002 | 0.028 | 0.020 |
|  |  | Number of different products available | 2 | 2 | 2 | 1 | 1 | 1 | 2 | 2 |
|  |  | Proportion of overall R03 consumption | 0.68% | 0.15% | 0.06% | 0.02% | 0.04% | 0.01% | 0.58% | 0.26% |
|  | R03AC13 formoterol  (LABA) | Cost of 1 DDD, US$ [mean (± 95%CI)] | $0.32 (CI±0.001) | $0.32 (CI±0.001) | $0.32 (CI±0.001) | $0.42 (CI±0.015) | $0.52 (CI±0.047) | … | $0.35 (CI±0.013) | $0.35 (CI±0.027) |
|  |  | Consumption in DID | 0.017 | 0.017 | 0.016 | 0.04 | 0.009 | … | 0.022 | 0.033 |
|  |  | Number of different products available | 1 | 1 | 1 | 1 | 1 | … | 2 | 1 |
|  |  | Proportion of overall R03 consumption | 0.13% | 0.13% | 0.11% | 0.16% | 0.06% | … | 0.37% | 0.41% |
|  | R03DC03  montelukast  (LTRA) | Cost of 1 DDD, US$ [mean (± 95%CI)] | $0.33 (CI±0.003) | $0.32 (CI±0.005) | $0.31 (CI±0.003) | $0.44 (CI±0.003) | $0.43 (CI±0.006) | $0.43 (CI±0.005) | $0.29 (CI±0.012) | $0.30 (CI±0.014) |
|  |  | Consumption in DID | 1.34 | 1.30 | 1.41 | 1.36 | 1.04 | 1.25 | 0.30 | 0.48 |
|  |  | Number of different products available | 37 | 34 | 34 | 45 | 53 | 45 | 44 | 53 |
|  |  | Proportion of overall R03 consumption | 16.9% | 16.2% | 15.1% | 8.2% | 8.5% | 8.9% | 18.9% | 20.9% |
|  | R03DA05  aminophylline  (xantines) | Cost of 1 DDD, US$ [mean (± 95%CI)] | $0.05 (CI±0.003) | $0.03 (CI±0.003) | $0.10 (CI±0.01) | $0.03 (CI±0.001) | $0.03 (CI±0.002) | $0.04 (CI±0.002) | $0.08 (CI±0.003) | $0.09 (CI±0.007) |
|  |  | Consumption in DID | 0,34 | 0,32 | 0,31 | 1,00 | 0,49 | 0,82 | 0,26 | 0,34 |
|  |  | Number of different products available | 7 | 5 | 3 | 4 | 6 | 5 | 7 | 7 |
|  |  | Proportion of overall R03 consumption | 13,5% | 11,9% | 12,6% | 17,4% | 22,6% | 17,8% | 31,3% | 33,6% |
|  | R03BA02  budesonide  (ICS) | Cost of 1 DDD, US$ [mean (± 95%CI)] | $0.82 (CI±0.045) | $0.81 (CI±0.02) | $0.72 (CI±0.05) | $0.96 (CI±0.006) | $1.24 (CI±0.031) | $1.21 (CI±0.043) | $0.45 (CI±0.026) | $0.72 (CI±0.092) |
|  |  | Consumption in DID | 0.24 | 0.23 | 0.47 | 1.00 | 0.49 | 0.82 | 0.03 | 0.07 |
|  |  | Number of different products available | 9 | 10 | 10 | 10 | 10 | 10 | 2 | 6 |
|  |  | Proportion of overall R03 consumption | 4.2% | 4.2% | 6.2% | 7.6% | 5.4% | 7.5% | 0.7% | 1.3% |
|  | R03BA05  fluticasone   (ICS) | Cost of 1 DDD, US$ [mean (± 95%CI)] | $1.60 (CI±0.595) | $0.56 (CI±0.29) | $1.48 (CI±0.128) | $0.91 (CI±0.024) | $1.20 (CI±0.021) | $1.28 (CI±0.027) | $0.27 (CI±0.078) | $0.42 (CI±0.022) |
|  |  | Consumption in DID | 0.07 | 0.17 | 0.06 | 0.11 | 0.07 | 0.07 | 0.09 | 0.11 |
|  |  | Number of different products available | 9 | 10 | 11 | 4 | 3 | 4 | 5 | 4 |
|  |  | Proportion of overall R03 consumption | 3.7% | 3.7% | 3.1% | 1.8% | 1.9% | 1.7% | 1.5% | 1.2% |

CI - confidence interval, SKU – stock-keeping unit (pack, tablet, vial, etc.), SABA - short acting beta-2 agonists, LABA - long-acting beta2-agonists, LTRA - leukotriene receptor antagonist, ICS - inhaled corticosteroids . “…” indicates that data were not available.

### Section 4: Medicines for cancer management

This section outlines medicines used for endocrine therapy in breast and prostate cancer treatment in outpatient settings.

Table 8. Comparison of trends in the consumption and pricing of medicines for hormone therapy for cancer in Azerbaijan, Georgia, and Uzbekistan.

| **Azerbaijan** | **Georgia** | **Uzbekistan** |
| --- | --- | --- |
| ***Medicines for cancer management in outpatient settings*** | | |
| Age-standardized deaths from prostate cancer: 6.03 per 100,000 in 2019. Age-standardized deaths from breast cancer: 9.84 per 100,000 in 2019.(1) | Age-standardized deaths from prostate cancer: 9.06 per 100,000 in 2019. Age-standardized deaths from breast cancer: 11.34 per 100,000 in 2019.(1) | Age-standardized deaths from prostate cancer: 4.06 per 100,000 in 2019. Age-standardized deaths from breast cancer: 9.74 per 100,000 in 2019.(1) |
| - *Anti-estrogens*: Irregular supply and high prices   The market share of anti-estrogens was the highest in Azerbaijan and accounted for more than 60% of the total consumption of medicines for endocrine therapy in 2019-21. Consumption was 5 times lower (2019-2020) than in Georgia.  Consumption fluctuated markedly: consumption was very low (likely reflecting a shortage in supply) in Q1 2019 and drops were seen in Q2 2020 (-40%), and Q3 2020 (-99%), followed by a return to consumption levels in Q2 2020 and then a jump in consumption by a factor of 4.5 in Q 2021.  The cost of 1 DDD for tamoxifen in Azerbaijan was significantly higher than in Georgia (2.8 times higher in 2019, 1.7 times higher in 2020, 2.4 times higher in 2021) and higher than in Uzbekistan (2.4 times higher in 2019).   - *Anti-androgens:* High fluctuation in consumption and low variability in price, with lower costs than in the other two countries   The market share of anti-androgens decreased from 8% in 2019 to 4% in 2021, with a reduction in the number of available products from 2 in 2019 to 1 in 2020-21, with only bicalutamide available. Consumption was highly variable, going from a decrease of 38% in Q1 2020 to a rise of 22% in Q2 2020, followed by a decrease of 16.9% in Q4 2020 and an increase of 29% in Q1 2021, and a fall of 39% in Q4 2021.  There was high variability in prices in 2019, with an increase of 65.7% in Q3 2019 followed by a fall of 39.7% in Q4 2019. There was little fluctuation in price following this (less than 0.5% quarter-on-quarter).  The cost of 1 DDD was 2 times higher in 2019 than in Georgia, and even after prices fell for bicalutamide, they remained higher than in Georgia (39% higher in 2020, 31% higher in 2021) and higher than in Uzbekistan (34% higher in 2020).   - Aromatase inhibitors: a growth in consumption in 2020-21 was accompanied by a 0.6% price reduction in 2021, however the cost of 1 DDD for anastrozole was 59% above the price in Georgia (2021) and 22% above the price in Uzbekistan (2020)   Aromatase inhibitors represented the second largest group of medicines for cancer management and their market share increased from 21% in 2019 to 32% in 2021, while the number of products doubled from 3 to 6, across 2 INNs (anastrozole and letrozole). As a result, consumption increased by a factor of 2.7 from 0.038 in 2019 to 0.102 in 2021.  Quarterly price fluctuation was low (less than 1%) and a sustained reduction in prices was seen in 2021.  The cost of 1 DDD for anastrozole was 59% higher than in Georgia (2021) and 22% higher than in Uzbekistan (2020)   - Gonadotropin releasing hormone analogues (GnRH)   There was high quarterly variation in consumption and prices, with a 54% increase in consumption in Q4 2020 accompanied by a decrease in prices of 6%, and a decrease in consumption by 44% in Q1 2021 accompanied by an increase in prices of 8.8% (Fig.8).  Prices of GnRH analogues was the highest in Azerbaijan compared to Georgia and Uzbekistan. The cost triptorelin in Azerbaijan 2.5 times higher than in Georgia in 2019, and 3.9 times higher in 2021. | - Anti-estrogens: Increases in consumption accompanying price reductions   Anti-estrogens: the market share decreased from 30% in 2019 to 19% in 2021 while the number of pharmaceutical remained the same through 2019-21, with 11 pharmaceutical products for 3 INNs.  There was moderate fluctuation in consumption (±30%) and decreases in prices, falling 32.6% in Q2 2019 and falling 76% in Q1 2021.   - *Anti-androgens*: there was moderate fluctuation in consumption and the lowest cost per DDD compared to the other two countries, despite annual growth in prices   The market share of anti-androgens decreased from 11.5% in 2019 to 9.8% in 2021, while the number of products remained the same: 3 products for 2 INNs (bicalutamide and flutamide).  There was moderate fluctuation in consumption (±20% quarter-on-quarter) along with price fluctuation at an average of ±10% quarter-on-quarter, although prices jumped by a factor of 2.5 in Q2 2020 followed by a decrease of 49% in Q3 2020, resulting in an average increase in price of 2.5 times in 2020 and a decrease of 44% in 2021.  The cost of 1 DDD of bicalutamide was lower than in Azerbaijan (2.5 times lower in 2019, 39% lower in 2020, 31% lower in 2021) and Uzbekistan (41% lower in 2019, 18% lower in 2020).   - Aromatase inhibitors: Consumption increased by a factor of 2.5, accompanied by a price increase of 58% in 2021 compared to 2019.   The market share of aromatase inhibitors is the largest among endocrine therapy for cancer, increasing from 42% in 2019 to 54% in 2021, representing 15 products for 3 INNs.  The consumption of aromatase inhibitors increased by 73% from 0.59 DID in 2019 to 1.02 DID in 2021.  Despite a rise in prices in Georgia, the cost of 1 DDD for anastrozole in Georgia was 60% lower than in Azerbaijan.   - Gonadotropin releasing hormone analogues (GnRH): The consumption of GnRH analogues increased in 2020-21 and was higher than in Azerbaijan and Uzbekistan. Despite a rise of 35% in price, the cost of 1 DDD was 3.9 times lower than in Azerbaijan (2021) and 2.4 times lower than in Uzbekistan (2020).   GnRH analogues included 15 products for 3 INNs. The market share increased from 15% to 16% as a proportion of consumption of medicines for endocrine therapy in cancer management, as well as increasing in terms of DID (from 0.264 DID in 2019 to 0.450 DID in 2021).  Goserelin accounted for the largest share (88% in 2020, 89% in 2021) of GnRH consumption despite a higher price compared to triptorelin (69% higher in 2020, 92% higher in 2021). | - Anti-estrogens: Shortages and irregular supply   Anti-estrogens: the market share of anti-estrogens was very small, compared to overall consumption of endocrine therapy for cancer, making up 2% in 2019 and 0.9% in 2020. Consumption of tamoxifen was limited to Q1 2019, and consumption of fulvestrant was limited to Q4 2019, and Q3-4 in 2020.  The cost of 1 DDD of tamoxifen was 2.4 times lower than in Azerbaijan but 16% higher than in Georgia in 2019.   - Anti-androgens: shortage and irregularity in supply   The market share of anti-androgens decreased from 6.9% in 2019 to 1.2% in 2020 and the number of pharmaceutical products decreased from 5 in 2019 to 3 in 2020, for 1 INN (bicalutamide). Sales of bicalutamide were limited to Q1-3 2019 and Q3-Q4 2020.  The cost of 1 DDD was lower than in Azerbaijan, by 17% in 2019 and by 26% in 2020, but higher than in Georgia, by 69% in 2019 and 22% in 2020.   - Aromatase inhibitors: Supply shortages and uneven supply, along with low consumption, were accompanied by an 86% price increase (letrozole)   Aromatase inhibitors made up the largest market share among medicines for endocrine therapy of cancer, but market share decreased from 72.1% in 2019 to 67.4% in 2020 and the number of pharmaceutical products decreased from 7 in 2019 to 5 in 2020, for 2 INNs.  Shortages in supply of both anastrozole and letrozole in Q1-Q2 2020 were accompanied by an 86% price increase in Q2-Q3 2020, followed by a 44% decrease in price at the end of 2020.  The cost of 1 DDD of letrozole was 18% lower than for anastrozole in 2019 and 28% lower in 2020, possibly reflecting consumer preference.   - Gonadotropin releasing hormone analogues (GnRH): shortages and supply disruptions were accompanied by a 17% price increase.   The consumption of GnRH analogues decreased 78% in 2020 compared to 2019, accompanied by high fluctuations in consumption and prices.  The cost of 1 DDD of triptorelin was 2.4 times higher than in Georgia and 75% higher than in Azerbaijan in 2020. |
| Figure 7. Consumption and price of triptorelin in Azerbaijan, Georgia, and Uzbekistan in 2019-2021 . | | |
|  |  |  |
| Figure 8. Cost of 1 DDD of medicines for cancer management in 3 selected countries, 2019-2021 or the latest available year, US$ | | |
|  |  |  |

Table 9. Trends in the consumption of medicines for cancer management, DID and cost of 1DDD by countries

| **Health condition** | **Medicine** | **Indicator** | **Azerbaijan** | | | **Georgia** | | | **Uzbekistan** | |
| --- | --- | --- | --- | --- | --- | --- | --- | --- | --- | --- |
|  |  |  | 2019 | 2020 | 2021 | 2019 | 2020 | 2021 | 2019 | 2020 |
| Breast cancer and prostate cancer | Endocrine therapy* | Consumption in DID | 0.19 | 0.20 | 0.32 | 1.64 | 1.98 | 2.30 | 0.06 | 0.02 |
|  |  | Number of different products available | 9 | 10 | 12 | 30 | 30 | 30 | 17 | 11 |
|  | Anti-estrogens (L02BA) | Consumption in DID | 0.132 | 0.122 | 0.193 | 0.621 | 0.630 | 0.65 | 0.002 | 0 |
|  |  | Number of different products available | 4 | 4 | 4 | 9 | 9 | 9 | 2 | 0 |
|  |  | Proportion of overall L02 consumption | 68% | 58% | 60% | 28% | 23% | 15% | 0.2% | 0% |
|  | Anti-androgens (L02BB) | Consumption in DID | 0.017 | 0.011 | 0.013 | 0.156 | 0.183 | 0.183 | 0.007 | 0.001 |
|  |  | Number of different products available | 2 | 1 | 1 | 3 | 3 | 3 | 5 | 3 |
|  |  | Proportion of overall L02 consumption | 8% | 5% | 4% | 11% | 11% | 10% | 6.9% | 1.2% |
|  | Aromatase inhibitors (L02BG) | Consumption in DID | 0.038 | 0.059 | 0.102 | 0.590 | 0.788 | 1.02 | 0.041 | 0.014 |
|  |  | Number of different products available | 3 | 5 | 6 | 15 | 15 | 15 | 7 | 5 |
|  |  | Proportion of overall L02 consumption | 21% | 29% | 32% | 42% | 46% | 54% | 72.1% | 67.4% |
|  | Gonadotropin releasing hormone analogues (L02AE) | Consumption in DID | 0.004 | 0.012 | 0.008 | 0.264 | 0.373 | 0.450 | 0.005 | 0.004 |
|  |  | Number of different products available | 3 | 3 | 4 | 12 | 12 | 12 | 10 | 8 |
|  |  | Proportion of overall L02 consumption | 3% | 8% | 4% | 18% | 20% | 20% | 20.6% | 28.9% |
|  | L02AE04  triptorelin | Cost of 1 DDD, US$ [mean (± 95%CI)] | $5.34 (CI±0.275) | $5.85 (CI±1.219) | $9.65 (CI±0.272) | $2.22 (CI±0.135) | $2.64 (CI±0.175) | $2.47 (CI±0.227) | $5.62 (CI±0.33) | $6.25 (CI±0.783) |
|  |  | Consumption in DID | 0.002 | 0.003 | 0.001 | 0.021 | 0.009 | 0.009 | 0.003 | 0.002 |
|  |  | Number of different products available | 2 | 2 | 2 | 4 | 4 | 4 | 3 | 2 |
|  |  | Proportion of overall L02 consumption | 1.6% | 2.8% | 2.1% | 3.0% | 1.1% | 0.9% | 5.6% | 17.6% |
|  | L02BA01  tamoxifen | Cost of 1 DDD, US$ [mean (± 95%CI)] | $0.16 (CI±0.002) | $0.14 (CI±0.015) | $0.19 (CI±0.001) | $0.05 (CI±0.003) | $0.08 (CI±0.004) | $0.07 (CI±0.003) | $0.06 (±0.065) | N/A |
|  |  | Consumption in DID | 0.094 | 0.073 | 0.143 | 0.406 | 0.324 | 0.390 | 0.0001 | N/A |
|  |  | Number of different products available | 2 | 2 | 2 | 7 | 7 | 7 | 2 | 0 |
|  |  | Proportion of overall L02 consumption | 48.6% | 34.6% | 44.1% | 23.6% | 19.3% | 13.2% | 0.2% | 0.0% |
|  | L02BB03  bicalutamide | Cost of 1 DDD, US$ [mean (± 95%CI)] | $0.61 (CI±0.018) | $0.62 (CI±0.002) | $0.62 (CI±0.001) | $0.29 (CI±0.009) | $0.38 (CI±0.028) | $0.43 (CI±0.029) | $0.50 (CI±0.016) | $0.46 (CI±0.013) |
|  |  | Consumption in DID | 0.017 | 0.011 | 0.013 | 0.152 | 0.171 | 0.167 | 0.007 | 0.001 |
|  |  | Number of different products available | 2 | 1 | 1 | 2 | 2 | 2 | 5 | 3 |
|  |  | Proportion of overall L02 consumption | 8.0% | 5.4% | 3.9% | 11.3% | 10.3% | 9.1% | 6.9% | 1.2% |
|  | L02BG03  anastrozole | Cost of 1 DDD, US$ [mean (± 95%CI)] | $0.73 (CI±0.012) | $0.81 (CI±0.029) | $0.89 (CI±0.013) | $0.41 (CI±0.004) | $0.55 (CI±0.023) | $0.56 (CI±0.011) | $0.65 (CI±0.109) | $0.67 (CI±0.096) |
|  |  | Consumption in DID | 0.032 | 0.050 | 0.083 | 0.342 | 0.540 | 0.644 | 0.004 | 0.004 |
|  |  | Number of different products available | 2 | 2 | 2 | 6 | 6 | 6 | 3 | 2 |
|  |  | Proportion of overall L02 consumption | 17.6% | 24.7% | 26.3% | 25.2% | 32.4% | 34.8% | 7.1% | 19.7% |

Notes: GnRH - Gonadotropin releasing hormone, SKU - stock keeping unit, DDD - defined daily dose, CI - confidence interval.
* ‘Endocrine therapy’ refers to the total across ATC class L02.

### Section 5: Medicines for epilepsy management

This section outlines medicines used epilepsy management under the N03 category of the ATC classification.

Table 10. Comparison of trends in the consumption and pricing of medicines for epilepsy in Azerbaijan, Georgia, and Uzbekistan.

| **Azerbaijan** | **Georgia** | **Uzbekistan** |
| --- | --- | --- |
| Age-standardized deaths from epilepsy: 2.02 per 100,000 in 2019.(1) | Age-standardized deaths from epilepsy: 0.58 per 100,000 in 2019.(1) | Age-standardized deaths from epilepsy: 3.33 per 100,000 in 2019.(1) |
| ***Medicines for epilepsy management in outpatient settings*** | | |
| During 2019-21, consumption of AEDs increased by 24%, from 1.30 to 1.61 DID, driven by an increase in consumption of second-generation AEDs: from 0.33 to 0.55 DID.  However, 1^st^-generation AEDs remain the most-consumed (making up 70.9% of AED consumption in 2021).  There were very low levels of consumption, possibly indicating shortages, for certain first-generation AEDs (clonazepam, phenobarbital).  There was moderate fluctuation in consumption and low fluctuation in prices, with the average price of valproate decreasing by 10% over 2019-21, and increased by 1% for carbamazepine. However, costs were higher in Azerbaijan than in Georgia: the cost of 1 DDD for carbamazepine was 27% higher in 2020 and 18% higher in 2021, the cost of valproate was 35% higher in 2020 and 27% higher in 2021. The cost of 1 DDD for valproate in Azerbaijan was 27% lower than in Uzbekistan, and for carbamazepine was 33% higher than in Uzbekistan, in 2020.  The market share of second-generation AEDs increased from 21.9% in 2019 to 29.1% in 2021, which the most-consumed being levetiracetam (12.1% in 2021) and pregabalin (8% in 2021).  There were very low levels of consumption, possibly indicating shortages, for second- and third-generation AEDs. | In 2020, coinciding with the start of the COVID-19 pandemic, the consumption of AEDs decreased by 38% from 4.38 to 2.70 DID, followed by an increase by 26% in 2021 by 26%, up to 3.40 DID.  Despite the reduction in the consumption of AEDs in 2019-21 in Georgia, consumption level remained at least twice that in Azerbaijan in 2021 and Uzbekistan in 2020. The most consumed were first-generation AEDs (83.3% of total AED consumption in 2021).  Consumption was the highest across the three countries, perhaps reflected in the lowest age-standardized death rate.(1)  There was moderate-to-high fluctuation in the consumption of first-generation AEDs, ranging from a drop of 33% in Q4 2019 to an increase of 17% in Q3 2020.  Over 2019-21, there were marked increases in prices of carbamazepine (39% over 2019-21) valproate (44% over 2019-21), with noticeable jumps in in Q3 2020 for carbamazepine and Q4 2020 for valproate, which coincided with the peak of the COVID-19 pandemic.  The cost of 1 DDD for the first-generation AEDs was lower than in Azerbaijan (for carbamazepine 27% lower, for valproate 35% lower) and lower than in Uzbekistan (valproate –52% lower), in 2020.  The market share of second-generation AEDs increased from 14.5% in 2019 to 16.7% in 2021. The most-consumed in 2021 was lamotrigine (8.7%), followed by levetiracetam (7.6%).  There were very low levels of consumption, possibly indicating shortages, for second- and third-generation AEDs. | Over 2019-2020, the consumption of AEDs reduced from 1.51 to 1.28 per 1000 people per day.  There was high fluctuation in the consumption of valproate (±50%) and low price fluctuation (±5%), while for carbamazepine prices jumped by 20% in Q2 2020, leading to an average 17% increase in price for carbamazepine and 6% increase in price for valproate in 2020 compared to 2019.  The market share for second-generation AEDs decreased from 25.2% in 2019 to 18.0% in 2020. The most- consumed AEDs were pregabalin in 2019 (15.2%) and gabapentin in 2020 (12%).  There were very low levels of consumption, possibly indicating shortages, for second-generation AEDs. |
| Figure 9. Consumption and price of metformin in Azerbaijan, Georgia, and Uzbekistan in 2019-2021 . | | |
|  |  |  |
|  |  |  |
| Figure 10. Cost of 1 DDD of medicines for epilepsy management in Azerbaijan, Georgia, and Uzbekistan, 2019-2021, US$ | | |
|  |  |  |
|  |  |  |

AZE = Azerbaijan, GEO - Georgia, UZB - Uzbekistan, DDD - defined daily dose, AED= Antiepileptic drugs.
For AEDs, drug categorization follows a generational pattern. First-generation AEDs refers to those drugs in use or approved for use before 1993, second-generation AEDs are those approved between 1993 and 2007 (e.g. pregabalin, lamotrigine, levetiracetam), and the most recent AEDs, approved after 2008, are referred to as third-generation agents (e.g. vigabatrin).

Table 11. Trends in the consumption and prices of medicines for epilepsy management in Azerbaijan, Georgia, and Uzbekistan, 2019-21.

| **Health condition** | **Medicine** | **Indicator** | **Azerbaijan** | | | **Georgia** | | | **Uzbekistan** | |
| --- | --- | --- | --- | --- | --- | --- | --- | --- | --- | --- |
|  |  |  | 2019 | 2020 | 2021 | 2019 | 2020 | 2021 | 2019 | 2020 |
| Epilepsy | Antiepileptic drugs (AED)^1^ | Consumption in DID | 1.30 | 1.50 | 1.61 | 4.38 | 2.70 | 3.40 | 1.51 | 1.28 |
|  |  | Number of different products available | 50 | 46 | 49 | 68 | 68 | 68 | 47 | 55 |
|  | AEDs, first generation | Consumption in DID | 0.98 | 1.07 | 1.07 | 3.62 | 2.22 | 2.73 | 1.00 | 1.02 |
|  |  | Number of different products available | 21 | 17 | 18 | 26 | 25 | 27 | 20 | 19 |
|  |  | Proportion of overall AED consumption | 78.1% | 72.6% | 70.9% | 85.4% | 84.0% | 83.3% | 74.8% | 82.0% |
|  | AEDs, second generation | Consumption in DID | 0.33 | 0.43 | 0.55 | 0.77 | 0.48 | 0.67 | 0.52 | 0.25 |
|  |  | Number of different products available | 29 | 29 | 31 | 41 | 42 | 40 | 27 | 36 |
|  |  | Proportion of overall AED consumption | 21.9% | 27.4% | 29.1% | 14.5% | 16.0% | 16.7% | 25.2% | 18.0% |
|  |  |  | N03AX14  levetiracetam | | | N03AX09  lamotrigine | | | N03AX12  gabapentin | |
|  |  |  | 11.0% | 9.4% | 12.1% | 7.5% | 7.0% | 8.7% | 7.6% | 12.0% |
|  | N03AF01  carbamazepine | Cost of 1 DDD, US$ [mean (± 95%CI)] | $0.30 (CI±0.004) | $0.29 (CI±0.002) | $0.29 (CI±0.002) | $0.18 (CI±0.001) | $0.21 (CI±0.002) | $0.24 (CI±0.003) | $0.23 (CI±0.013) | $0.22 (CI±0.016) |
|  |  | Consumption in DID | 0.70 | 0.77 | 0.70 | 2.43 | 1.38 | 1.69 | 0.73 | 0.72 |
|  |  | Number of different products available | 7 | 6 | 6 | 13 | 12 | 14 | 8 | 7 |
|  |  | Proportion of overall N03 consumption | 54.7% | 50.8% | 46.2% | 55.3% | 51.0% | 49.1% | 60.8% | 64.0% |
|  | N03AG01  valproic acid | Cost of 1 DDD, US$ [mean (± 95%CI)] | $0.63 (CI±0.003) | $0.60 (CI±0.002) | $0.61 (CI±0.009) | $0.32 (CI±0.007) | $0.39 (CI±0.006) | $0.44 (CI±0.019) | $0.89 (CI±0.006) | $0.82 (CI±0.013) |
|  |  | Consumption in DID | 0.22 | 0.25 | 0.27 | 0.80 | 0.55 | 0.76 | 0.21 | 0.23 |
|  |  | Number of different products available | 11 | 8 | 8 | 6 | 6 | 6 | 11 | 11 |
|  |  | Proportion of overall N03 consumption | 19.6% | 18.7% | 19.8% | 18.9% | 20.3% | 23.2% | 10.3% | 12.8% |

AED= Antiepileptic drugs. For AEDs, drug categorization follows a generational pattern. First-generation AEDs refers to those drugs in use or approved for use before 1993, second-generation AEDs are those approved between 1993 and 2007 (e.g. pregabalin, lamotrigine, levetiracetam), and the most recent AEDs, approved after 2008, are referred to as third-generation agents (e.g. vigabatrin).

### Section 6: Medicines for mental disorders management

This section outlines medicines used in the treatment of anxiety and depressive disorders, under the N05B and N06A categories of the ATC classification.

Table 12. Comparison of trends in the consumption and pricing of medicines for mental health disorders in Azerbaijan, Georgia, and Uzbekistan.

| **Azerbaijan** | **Georgia** | **Uzbekistan** |
| --- | --- | --- |
| Prevalence of depression: 3.13% in 2019. Prevalence of anxiety disorders: 2.75% in 2019.(1) | Prevalence of depression: 5.08% in 2019. Prevalence of anxiety disorders: 6.57% in 2019.(1) | Prevalence of depression: 3.12% in 2019. Prevalence of anxiety disorders: 2.11% in 2019.(1) |
| ***Medicines for mental disorders management in outpatient settings*** | | |
| *Anxiety disorders*  The consumption of medicines for anxiety disorders treatment decreased from 0.86 DID in 2019 to 0.77 DID in 2021.  Consumption was high variable from quarter to quarter.  The most consumed anxiolytic was tofisopam (representing 43.0% of all anxiolytics in 2019), whose market share declined to 26.5% in 2020 but increased to 57.6% in 2021. However, consumption of tofisopam per 1000 inhabitants per day was 4 times lower than in Georgia.  The market share of hydroxyzine increased in 2020 from 51.2% to 67.6%, followed by a decrease to 30.4% in 2021.  The price of hydroxyzine reduced by 10% over 2019-21, while tofisopam price showed minimal changes quarter-on-quarter (±1%).  The cost of 1 DDD for tofisopam remained at the same level ($0.13) during 2019-2021, but was 26% higher than in Georgia, while the cost of 1 DDD for hydroxyzine was the same as in Georgia in 2021 ($0.23).  *Depressive disorders*  The consumption of antidepressants increased by 31% in 2019-2021, from 5.00 to 6.57 DID. However, the consumption of antidepressants in Azerbaijan in 2021 was 10 times lower than the average across OECD countries (66.0 DID) and 6.7 times lower than in Turkey (44.0 DID) in 2019.  The most consumed antidepressant was escitalopram, with its market share increasing from 38.3% in 2019 to 39.3% in 2021, while the market share of amitriptyline decreased from 17.9% to 16.2% over the same period.  The price of escitalopram decreased by 2% and prices fluctuated little (±2% quarter-on-quarter), while the amitriptyline had pronounced price fluctuations, with a jump of 43% in Q2 2020 and a jump of 65% in Q1 2021. High fluctuation in prices for amitriptyline can be explained by the supply of imported medicines, as prices for locally produced versions remained at the same lower level (Fig 11).  The cost of 1 DDD of escitalopram ($0.22) was 79% lower than in Georgia ($0.40) in 2021 but 6% higher than in Uzbekistan ($0.21) in 2020. At the same time, the cost of 1 DDD of amitriptyline ($0.06) was 41% lower than in Uzbekistan ($0.08) in 2020 and 70% lower than in Georgia ($0.10) in 2021 (table 13). | *Anxiety disorders*  The consumption of anxiolytics declined by 6% in 2019-2020, from 6.10 to 5.72 DID, followed by an increase of 18% in 2021, to 6.73 DID.  The consumption of medicines for anxiety disorders in Georgia was 9 times higher than in Azerbaijan in 2021 and 11.6 times higher than in Uzbekistan in 2020.  The anxiolytic with the highest consumption was hydroxyzine, although its market share decreased from 37% in 2019 to 33% in 2021, while the market share of tofisopam increased from 22% to 27% over the same period.  The cost of 1 DDD of hydroxyzine increased by 34% over 2019-21 and was almost 3 times above the cost of 1 DDD for tofisopam in 2021.  *Depressive disorders*  Georgia had the highest levels of antidepressant consumption, 26% higher than in Azerbaijan and 6.7 times higher than in Uzbekistan. The consumption of antidepressants declined by 12% in 2020, followed by an increase of 20% in 2021.  The most consumed antidepressant was escitalopram, although its market share decreased slightly, from 26.0% in 2019 to 25.9% in 2021, while the market share of amitriptyline decreased from 17.9% to 16.2% over the same period. Growth in consumption was accompanied with a steady rise in prices, with the price of escitalopram increasing by 37% and the price of amitriptyline increasing by 60% over 2019-21 (Fig 11).  The cost of 1 DDD of escitalopram was higher than in Azerbaijan (by 79% in 2021) and Uzbekistan (by 75% in 2020), while the cost of 1 DDD of amitriptyline was 23% lower than in Uzbekistan but 14% higher than in Azerbaijan, in 2020 (table 13). | *Anxiety disorders*  The consumption of anxiolytics reduced by 13% over 2019-21, from 0.57 to 0.49 DID. There was high quarter-to-quarter price fluctuation (>30%)(Fig.11).  The most consumed anxiolytic was tofisopam in 2020, with market share increasing from 26% in 2019 to 34% in 2020 despite the very low consumption in Q4 2019 and Q3-Q4 2020. The market share of diazepam reduced from 40% in 2019 to 32% in 2020 (table 13).  The price of tofisopam doubled in 2020 (Fig.11).  The price of hydroxyzine was 67% lower than the price of tofisopam in 2020, while the cost of 1 DDD of tofisopam was 5% above the price of diazepam.  Very low levels of consumption were seen for hydroxyzine in Q4-2019-Q2 2020, possibly indicating shortages. This was accompanied by a 20% increase in price in Q2 2020, followed by a 15% decrease in Q4 2020 (Fig 11).  The cost of 1 DDD of tofisopam was almost 3 times the cost in Georgia and 1.5 times the cost in Azerbaijan, in 2020 (Fig. 11, table 13)  *Depressive disorders*  There were very low levels of consumption of antidepressants, which decreased from 1.30 DID in 2019 to 1.07 DID in 2020. The largest drop was seen in 4Q 2019, with a drop in consumption of a factor of 3.8, followed by continuous growth in Q1-Q3 2020, with consumption in Q3 2020 1.7 times higher than in Q1 2020.  The most consumed antidepressant in 2020 was amitriptyline, whose market share increased from 48% in 2019 to 53% in 2020. The consumption of amitriptyline increased slightly, from 0.41 to 0.42 DID. A 35% increase in prices for amitriptyline was seen in Q2-Q4 2020, overall the price increased by 13% over 2019-20 (Fig 11).  The consumption of escitalopram jumped by 73% in Q3 2020, with price rising by 9% in Q2 2020 (Fig 11).  The cost of 1 DDD of amitriptyline in 2020 was 41% higher than in Azerbaijan and 23% higher than in Georgia, but 2.6 times lower than the cost of 1 DDD of escitalopram, while the cost of 1 DDD of escitalopram in Uzbekistan was 43% lower than in Georgia (table 13). |
| Figure 11. Consumption and price of tofisopam in Azerbaijan, Georgia, and Uzbekistan in 2019-2021. | | |
|  |  |  |
|  |  |  |
| Figure 12. Cost of 1 DDD of medicines for mental health disorders in Azerbaijan, Georgia, and Uzbekistan, 2019-2021, US$. | | |
|  |  |  |
| Figure 13. Consumption and prices of antidepressants in Azerbaijan, Georgia, and Uzbekistan in 2019-2021. | | |
|  |  |  |
|  |  |  |

Note: 1. AZE - Azerbaijan, GEO - Georgia, UZB - Uzbekistan, DDD - defined daily dose, CI - confidence interval, SKU - stock keeping unit.

Sources for consumption and epidemiological data: *2.* OECD, 2022 (3)*,* IHME, 2022 (2)

Table 13. Trends in the consumption of medicines for mental disorders management in Azerbaijan, Georgia, and Uzbekistan, 2019-21.

| **Health condition** | **Medicine** | **Indicator** | **Azerbaijan** | | | **Georgia** | | | **Uzbekistan** | |
| --- | --- | --- | --- | --- | --- | --- | --- | --- | --- | --- |
|  |  |  | 2019 | 2020 | 2021 | 2019 | 2020 | 2021 | 2019 | 2020 |
| Anxiety disorders | Anxiolytics (N05B) | Consumption in DID | 0.86 | 0.85 | 0.77 | 6.10 | 5.72 | 6.73 | 0.57 | 0.49 |
|  |  | Number of different products available | 8 | 8 | 8 | 34 | 36 | 34 | 15 | 15 |
|  | N05BB01 hydroxyzine | Cost of 1 DDD, US$ [mean (± 95%CI)] | $0.26 (CI±0.05) | $0.22 (CI±0.001) | $0.22 (CI±0.003) | $0.17 (CI±0.003) | $0.20 (CI±0.006) | $0.23 (CI±0.007) | $0.43 (CI±0.067) | $0.40 (CI±0.026) |
|  |  | Consumption in DID | 0.27 | 0.41 | 0.12 | 1.28 | 0.99 | 1.15 | 0.053 | 0.058 |
|  |  | Number of different products available | 1 | 1 | 1 | 4 | 4 | 4 | 4 | 4 |
|  |  | Proportion of overall anxiolytic (N05) consumption | 51.2% | 67.6% | 30.4% | 36.7% | 32.1% | 33.4% | 15.0% | 19.8% |
|  | N05BA23 tofisopam | Cost of 1 DDD, US$ [mean (± 95%CI)] | $0.12 (CI±0.002) | $0.12 (CI±0.001) | $0.12 (CI±0.002) | $0.05 (CI±0.002) | $0.06 (CI±0.003) | $0.07 (CI±0.006) | tofisopam $0.16 (CI±0.016) | tofisopam $0.20 (CI±0.01) |
|  |  |  |  |  |  |  |  |  | diazepam  $0.14 (CI±0.041) | diazepam  $0.19 (CI±0.088) |
|  |  | Consumption in DID | 0.54 | 0.38 | 0.56 | 1.86 | 1.80 | 2.26 | tofisopam 0.195 | tofisopam 0.190 |
|  |  |  |  |  |  |  |  |  | diazepam 0.10 | diazepam 0.05 |
|  |  | Number of different products available | 1 | 1 | 1 | 1 | 1 | 1 | tofisopam 1 | tofisopam 2 |
|  |  |  |  |  |  |  |  |  | diazepam 4 | diazepam 3 |
|  |  | Proportion of overall anxiolytic (N05) consumption | 43.0% | 26.5% | 57.6% | 22.0% | 24.2% | 27.2% | tofisopam 22.7% | tofisopam 24.4% |
|  |  |  |  |  |  |  |  |  | diazepam  40.4% | diazepam  31.9% |
| Depressive disorders | Antidepressants (N06A) | Consumption in DID | 5.00 | 5.67 | 6.57 | 8.13 | 7.16 | 8.61 | 1.30 | 1.07 |
|  |  | Number of different products available | 68 | 63 | 66 | 77 | 78 | 81 | 42 | 42 |
|  | N06AB10 escitalopram | Cost of 1 DDD, US$ [mean (± 95%CI)] | $0.23 (CI±0.001) | $0.22 (CI±0.003) | $0.22 (CI±0.002) | $0.32 (CI±0.002) | $0.37 (CI±0.004) | $0.39 (CI±0.005) | $0.20 (CI±0.006) | $0.21 (CI±0.015) |
|  |  | Consumption in DID | 2.47 | 2.85 | 3.29 | 2.57 | 2.42 | 2.90 | 0.22 | 0.15 |
|  |  | Number of different products available | 2 | 2 | 2 | 23 | 26 | 28 | 5 | 6 |
|  |  | Proportion of overall anxiolytic (N05) consumption | 38.3% | 38.6% | 39.3% | 26.0% | 27.4% | 25.9% | 10.8% | 7.3% |
|  | N06AA09  amitriptyline | Cost of 1 DDD, US$ [mean (± 95%CI)] | $0.05 (CI±0.001) | $0.05 (CI±0.001) | $0.05 (CI±0.001) | $0.05 (CI±0.001) | $0.06 (CI±0.002) | $0.09 (CI±0.008) | $0.07 (CI±0.003) | $0.08 (CI±0.003) |
|  |  | Consumption in DID | 0.57 | 0.66 | 0.69 | 0.75 | 0.58 | 0.60 | 0.41 | 0.42 |
|  |  | Number of different products available | 1 | 1 | 1 | 5 | 4 | 4 | 9 | 10 |
|  |  | Proportion of overall anxiolytic (N05) consumption | 17.9% | 17.7% | 16.2% | 15.3% | 13.4% | 11.8% | 47.7% | 52.6% |

Note: N05B – Anxiolytics, N06A antidepressants, DDD=defined daily dose, SDR=standardised death rate, CI=confidence interval, SKU=stock keeping unit

**References**

1. Vos T, Lim SS, Abbafati C, Abbas KM, Abbasi M, Abbasifard M, et al. Global burden of 369 diseases and injuries in 204 countries and territories, 1990–2019: a systematic analysis for the Global Burden of Disease Study 2019. The Lancet. 2020 Oct;396(10258):1204–22.

2. Institute for Health Metrics and Evaluation. GBD Results Tool [Internet]. [cited 2017 Aug 4]. Available from: http://ghdx.healthdata.org/gbd-results-tool

3. Pharmaceutical consumption. In: Health at a Glance 2021 [Internet]. OECD; 2021 [cited 2022 Nov 30]. (Health at a Glance). Available from: https://www.oecd-ilibrary.org/social-issues-migration-health/health-at-a-glance-2021_5689c05c-en
